# Survival Rate and Chronic Diseases of TCGA Cancer and KoGES Normal Samples by Clustering for DNA Methylation

**DOI:** 10.3390/life14060768

**Published:** 2024-06-17

**Authors:** Jeong-An Gim

**Affiliations:** Department of Medical Science, Soonchunhyang University, Asan 31538, Republic of Korea; vitastar@sch.ac.kr; Tel.: +82-41-530-4707

**Keywords:** TCGA, KoGES, DNA methylation, survival rate, complex diseases

## Abstract

Insights from public DNA methylation data derived from cancer or normal tissues from cancer patients or healthy people can be obtained by machine learning. The goal is to determine methylation patterns that could be useful for predicting the prognosis for cancer patients and correcting lifestyles for healthy people. DNA methylation data were obtained from the DNA of 446 healthy participants from the Korean Genome Epidemiology Study (KoGES) and from the DNA of normal tissues or from cancer tissues of 11 types of carcinomas from The Cancer Genome Atlas (TCGA) database. To correct for the batch effect, R’s ComBat function was used. Using the K-mean clustering (k = 3), the survival rates of the cancer patients and the incidence of chronic diseases were compared between the three clusters for TCGA and KoGES, respectively. Based on the public DNA methylation and clinical data of healthy participants and cancer patients, I present an analysis pipeline that integrates and clusters the methylation data from the two groups. As a result of clustering, CpG sites from gene or genomic regions, such as AFAP1, NINJ2, and HOOK2 genes, that correlated with survival rate and chronic disease are presented.

## 1. Introduction

The Cancer Genome Atlas (TCGA) is a cancer genome database that provides information on variants, copy number variations (CNVs), RNA sequencing (RNA-seq), small RNA-seq (sRNA-seq), and DNA methylation for a total of 33 carcinomas. To understand the genetic patterns that cause cancer, TCGA aims to provide clues that can diagnose, treat, and prevent cancer [1,2]. TCGA was a pilot project for three years in 2006, and omics data were analyzed in three carcinomas (glioblastoma, lung cancer, and ovarian cancer), and omics data for a total of 33 carcinomas, including 10 rare cancers, were accumulated from 2009 to 2014. The data have been completed and open to the public. By secondary analysis of the published data, it was possible to have a deep understanding according to the subtype and histological patterns of cancer [1,3,4].

The Korean Genome and Epidemiology Study (KoGES) is a cohort study to find risk factors for chronic diseases in Koreans. Among them, the Ansung–Ansan cohort was started in 2001 for the general population aged 40–60, and follow-up was conducted every 2 years after the base survey of 10,030 people [5]. Participants filled out a questionnaire to check their current health status and lifestyle, and they received a health check-up. Samples of blood (30 mL) and urine (15 mL) were collected and used for clinical examination. A genotype analysis was performed on a total of 8840 people in the baseline survey [6,7], and metabolite analysis was performed on 2580 people in the second follow-up after 4 years of the baseline survey [8]. DNA methylation analysis was performed on 446 people in the baseline survey, and additional analysis was performed on 50 out of 446 people in the fourth follow-up after 8 years to observe changes in DNA methylation patterns according to time and lifestyle [9,10].

DNA methylation is a biological process in which a methyl group is added to the 5th carbon of cytosine constituting DNA. It is an epigenetic mechanism that induces biological changes without sequence change [11]. DNA methylation generally plays a role in suppressing the overexpression of genes by hypermethylation. DNA methylation is known to be involved in development, X chromosome imprinting, aging, and carcinogenesis. DNA methylation varies with sex, age, disease state, and lifestyle [12,13]. By dietary intervention before and after clinical treatment, a time-dependent change in DNA methylation according to lifestyle was reported [14,15]. The degree of DNA methylation is presented as a beta value, which is a value between 0 and 1, and the closer to 1, the more CpG methylation occurs. DNA methylation patterns differ according to sex, because women have two X chromosomes, so genomic imprinting occurs in one of them. This shows higher methylation levels in females, which is a reason to analyze females and males separately [9,15].

DNA methylation patterns are modified by aging, and epigenetic clocks were revealed from the normal participants as well as the KoGES sample in the general population aged 40–60 years [15,16,17]. In general, cancer often develops after the age of 50, so this study aims to find clustering patterns of DNA methylation in normal and cancer subjects. For clustering on the degree of DNA methylation, cancer patients and normal participants were merged. Three clusters were presented in two cohorts, and survival analysis for a total of six groups and the presence or absence of chronic disease were confirmed. A confusion matrix-based statistical process was provided for survival and chronic disease for each of the three clusters based on DNA methylation. The merging and clustering of data from TCGA and the KoGES is attempted for the first time in this study, presenting a challenging method of utilizing public omics data.

## 2. Materials and Methods

### 2.1. Data Acquisition of TCGA

**Figure 1:**
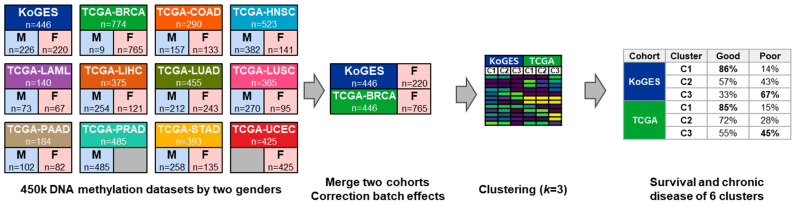
Processes of this study. The number of samples analyzed by 450 k DNA methylation was presented separately for KoGES normal participants and two sexes of each cancer type. DNA methylation information between normal participants and cancer patients was merged, and after clustering, genes with large standard deviations for each gene were selected. In each cluster presented as an example, the highest good and poor ratios for each cohort are displayed in bold. In KoGES, poor is considered chronic disease, and in TCGA, it is considered dead.

The overall process of this study is presented in Figure 1. To download the DNA methylation dataset stored in TCGA, the GDCquery function included in R’s TCGAbiolinks package was used [18]. The R version used was 4.1.2. The parameters used when using the GDCquery function are as follows: The project used the project names for each of the 11 cancer types shown in Figure 1 including TCGA-BRCA; the data.category is “DNA Methylation”; the data.type is “Methylation Beta Value”; the workflow.type is “Liftover”. Samples using the Infinium HumanMethylation450 BeadChip (Infinium 450k chip, Illumina, San Diego, CA, USA) were used, and the number of male and female samples is shown in Figure 1.

### 2.2. Data Acquisition of KoGES

The epidemiologic and DNA methylation analysis dataset used in this study was obtained from the Ansung–Ansan cohort of the KoGES surveyed between 2001 and 2002. Participants were between 40 and 69 years of age and resided in Ansung-si or Ansan-si, Gyeonggi-do, Republic of Korea. All participants provided written informed consent. For the DNA methylation dataset (Infinium 450k chip), a matrix for 446 people provided by the KoGES was used. Then, the bioinformatic analysis process was carried out as described in previous studies [9,10,15]. Most participants of the KoGES were healthy, but some had cancer or chronic diseases. KoGES datasets can be obtained by following the procedures set by the Ministry of Health and Welfare of the Republic of Korea. This study was conducted in accordance with the Declaration of Helsinki with the approval of Korea University IRB (Institutional Review Board) (approval number: KUIRB-2020-0191-01).

### 2.3. Selecting Dataset, Merging, and Clustering

To correct for the batch effect on the DNA methylation dataset obtained from two different cohorts, the “ComBat” function included in the “sva” package of R was used [19]. To avoid bias due to differences in DNA methylation between sexes, the two sexes were separated [9]. The integration of the two datasets used the R default “merge” function, and the index was the probe ID used in the Infinium 450k chip. To annotate the gene symbol corresponding to each probe ID, the “getAnnotation” function of the “IlluminaHumanMethylation450kanno.ilmn12.hg19” package was used.

Each DNA methylation level for the sample was visualized and clustered using R’s pheatmap package. To designate the k value in the k-means clustering algorithm, the value of “cutree_col” among the parameters of the “pheatmap” function was set to 3.

### 2.4. Visualization

For TCGA-derived carcinoma, follow-up data and mortality data were included, and a Kaplan–Meier (K-M) survival plot was drawn based on these data. Survival analysis was performed using the “survfit” function in the survival package of R, and a K-M plot was presented using the “ggsurvplot” function in the survminer package.

## 3. Results

The flowchart of this study is presented in Figure 1. A total of 11 datasets were used in this study. The tumor types are listed in Table S1.

### 3.1. Clustering DNA Methylation Levels

For a total of 11 carcinomas, a total of 19 clusterings was performed by sex. For each result, the DNA methylation level and cluster were provided simultaneously as a heatmap. Survival analysis in TCGA was presented, and a confusion matrix according to chronic disease was presented.

**Figure 2:**
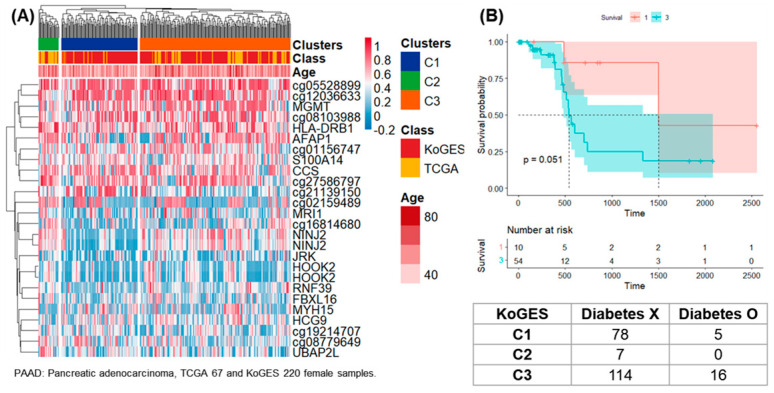
Clustering, survival analysis, and confusion matrix of female DNA methylation. (**A**) The clustering results of 67 female samples from TCGA-PAAD and 220 female samples from KoGES were presented as heatmaps. (**B**) Kaplan–Meier plots for TCGA-PAAD patients in clusters 1 and 3 (unit: day). Confusion matrix according to diabetes status for 220 women in KoGES.

Among them, clustering results were presented for samples derived from 67 women from TCGA-PAAD and from 220 women from KoGES (Figure 2). Overall, 26 DNA methylation analysis results satisfying standard deviation (SD) > 0.22 for each CpG site were presented as a heatmap and three clusters. Among the 287 samples, the number of samples corresponding to clusters 1, 2, and 3 was 93, 25, and 184 (Figure 2A).

**Figure 3:**
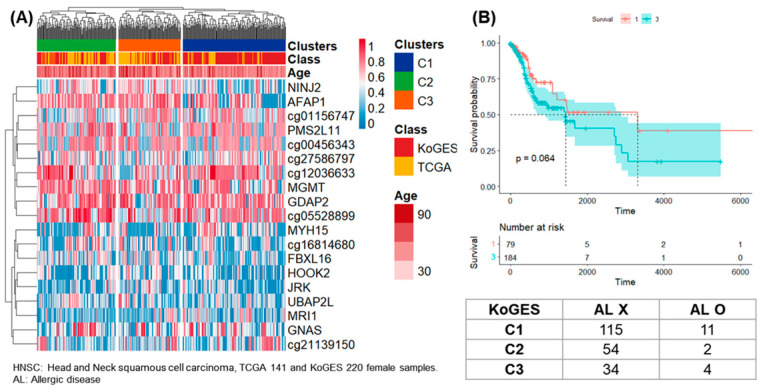
Clustering, survival analysis and confusion matrix of female DNA methylation. (**A**) The clustering results of 141 female samples from TCGA-HNSC and 220 female samples from KoGES are presented as heatmaps. (**B**) Kaplan–Meier plots for TCGA-HNSC patients in clusters 1 and 3 (unit: day). Confusion matrix according to allergic diseases in 220 women of KoGES.

The results of clustering of samples from 141 females in TCGA-HNSC and 220 females in the KoGES were presented (Figure 3). By DNA methylation analysis, 19 CpG sites satisfying SD > 0.44 were presented as a heatmap and three clusters. Of the total 361 samples, 152, 117, and 92 were classified as clusters 1, 2, and 3.

**Figure 4:**
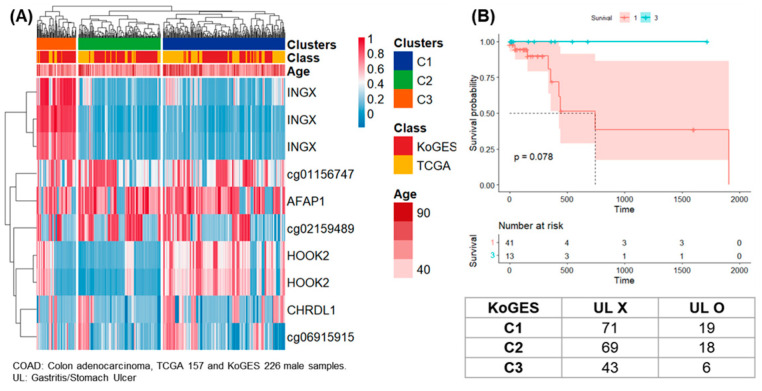
Clustering, survival analysis, and confusion matrix of male DNA methylation. (**A**) The clustering results of 157 male samples from TCGA-COAD and 226 male samples from KoGES are presented as heatmaps. (**B**) Kaplan–Meier plots for TCGA-COAD patients in clusters 1 and 3 (unit: day). Confusion matrix according to the presence of gastritis or stomach ulcer in 226 men of KoGES.

The clustering of samples from 157 males of TCGA-COAD and 226 males of KoGES were presented (Figure 4). The results satisfying SD > 0.25 for 10 CpG sites were presented as a heatmap and three clusters. Of the total 383 samples, 192, 129, and 62 were classified as clusters 1, 2, and 3.

**Figure 5:**
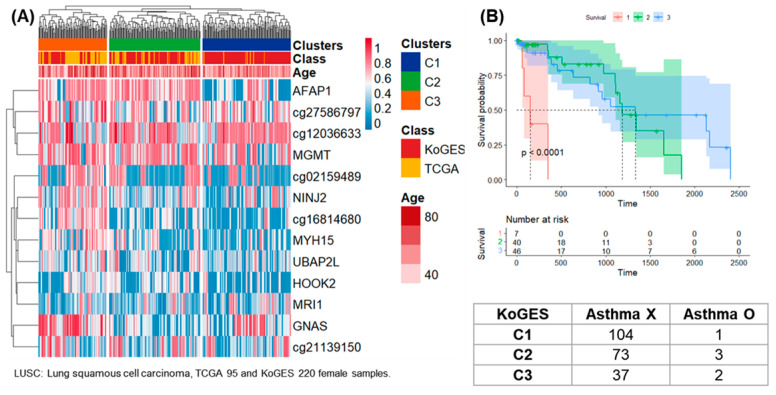
Clustering, survival analysis, and confusion matrix of female DNA methylation. (**A**) The clustering results of 95 female samples from TCGA-LUSC and 220 female samples from KoGES are presented as heatmaps. (**B**) Kaplan–Meier plots for TCGA-LUSC patients in clusters 1, 2 and 3 (unit: day). Confusion matrix according to the presence or absence of asthma in 220 women of KoGES.

The results of clustering of samples from 95 females of TCGA-LUSC and 220 females of KoGES are presented in Figure 5. The results of 13 CpG sites by DNA methylation analysis satisfying SD > 0.25 were presented as a heatmap and three clusters. Of the total 315 samples, 112, 116, and 87 were classified as clusters 1, 2, and 3.

**Figure 6:**
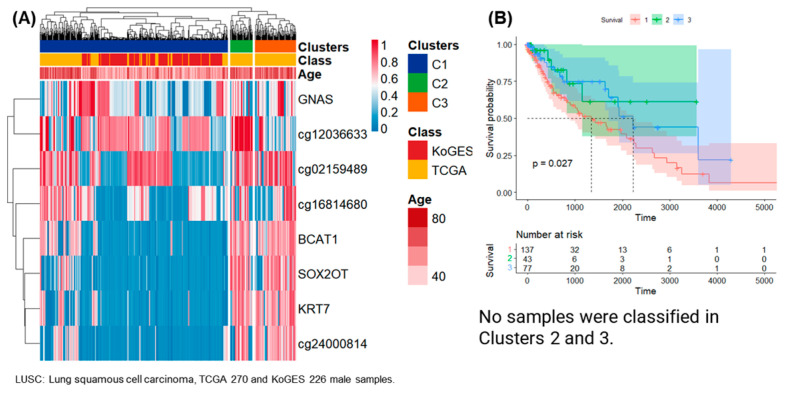
Clustering, survival analysis, and confusion matrix of male DNA methylation. (**A**) The clustering results of 270 male samples from TCGA-LUSC and 226 male samples from KoGES are presented as heatmaps. (**B**) Kaplan–Meier plots for TCGA-LUSC patients in clusters 1, 2 and 3 (unit: day). The confusion matrix for 220 men in KoGES was classified by chronic disease, but clustering was not provided.

The clustering results of 270 male samples from TCGA-LUSC and 226 male samples from the KoGES are presented in Figure 6. Eight DNA methylation analysis results satisfying SD > 0.29 for each CpG site were presented as a heatmap and three clusters. Overall, 371, 44, and 81 of a total of 496 samples were classified as clusters 1, 2, and 3. In the case of 226 samples of the KoGES, only cluster 1 was classified.

### 3.2. Survival Analysis

Survival analysis was performed on clusters 1 and 3 in female samples of TCGA-PAAD, and 10 patients in cluster 1 had a higher 5-year follow-up survival rate than 54 patients in group 3 (Figure 2B). Survival analysis was performed on clusters 1 and 3 in female samples of TCGA-HNSC, and 79 patients in cluster 1 had a higher 5-year follow-up survival rate than 184 patients in cluster 3 (Figure 3B). Survival analysis was performed for clusters 1 and 3 in male samples of TCGA-COAD, and all 13 patients included in cluster 3 survived follow-up. On the other hand, about half of the 41 patients included in cluster 1 survived for about 2 years (Figure 4B). Survival analysis was performed for the entire cluster in female samples of TCGA-LUSC. As a result, seven samples belonging to cluster 1 showed the lowest survival rate (Figure 5B). Survival analysis was performed on the whole cluster in male samples of TCGA-LUSC. As a result, a relatively low survival rate was confirmed in 137 samples belonging to cluster 1 (Figure 6B).

### 3.3. Confusion Matrix Analysis

Chronic disease was checked for KoGES samples belonging to the same cluster as TCGA-PAAD. Among the 13 chronic diseases, the odds ratio was 2.18 between clusters 1 and 3 for factors related to diabetes (Figure 2). This means that the prevalence of diabetes in the general population included in cluster 3 is higher. In the survival analysis of TCGA, the patients belonging to cluster 3 had a poor survival rate, so the pattern corresponding to cluster 3 can be considered a worse pattern than that of cluster 1. In female samples of the KoGES, which belonged to the same cluster as TCGA-HNSC, the presence of chronic disease in clusters 1 and 3 was confirmed. The odds ratio for allergic disease was 1.23. In cluster 3, the allergic disease rate was high, but there was no statistical significance (Figure 3).

### 3.4. Commonly Related Genes by Venn Diagram of Five Clustering Datasets

**Figure 7:**
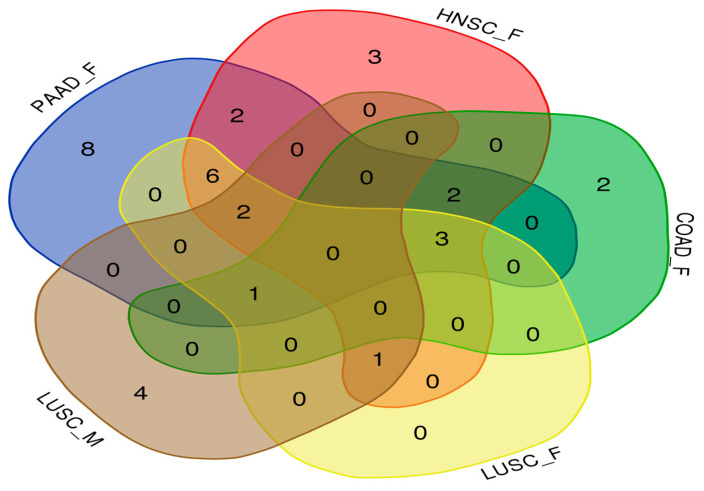
Venn diagram of five analyses.

The Venn diagrams for the genes detected in the five clustering datasets are presented (Figure 7). Commonly detected genes are listed in Table 1, and accession numbers were provided when the CpG sites were not located in the gene regions. A total of six CpG sites were commonly detected in the four clustering datasets, and all were analyzed in females. Nine CpG sites were detected in three assays, and all were analyzed in females, except for LUSC male assays. In female analysis of PAAD, a total of eight CpG sites were specifically identified.

## 4. Discussion

Currently, cohort-based DNA methylation data have been accumulated from various samples, such as the bloods of normal participants, bloods of cancer patients, and cancer tissues. For one individual, a comparative study between baseline and follow-up according to lifestyle was performed, and analysis methods were presented [5,15]. In this study, DNA methylation data of TCGA cancer patients and healthy normal subjects obtained from KoGES were merged. I tried to find insights for prediction factors of cancer prognosis and chronic diseases from DNA methylation dataset, so I provided novel methods of DNA methylation data by merging and clustering.

After the multi-omics data of TCGA was published, modeling approaches have been conducted to classify normal and cancer or each subtype of cancer [20,21,22]. Colorectal cancer susceptibility using 845 DNA methylation data from GSE51032 of “the Italian arm of the European Prospective Investigation into Cancer and Nutrition (EPIC-Italy)”, a cohort study conducted in Italy, was presented. Comparative verification was performed in TCGA based on the results of GSE51032 [23]. Based on the results of TCGA-BRCA DNA methylation analysis, the GO terms and pathways of 267 differentially methylated regions (DMRs) were discovered. For high- and low-risk classification, a model based on the DNA methylation level of QRFP, S100A16, TDRD1, and SMO genes was presented. Negative correlations for 10 genes were presented, and survival rates were provided for patients classified as high and low risk [24]. In these studies, DNA methylation patterns of cancer that were different from normal subjects were revealed.

The meta-analysis of cancer markers related to DNA methylation was performed. Koch et al. reported the meta-analysis results in 2018. In that paper, 1800 markers were presented in 14,743 research articles [25], but only 14 of them were actually used commercially. Each gene was characterized as a biomarker based on DNA methylation for GSTP1, APC, RASSF1, NDRG2, BMP3, SEPT9, SHOX2, TWIST1, OTX1, ONECUT2, MGMT, BCAT1, and IKZF1 genes. In this study, a Venn diagram was presented for genes detected at least once in a total of five clustering datasets (Figure 7), and common MGMT and BCAT1 genes were identified. The MGMT gene was detected in female HNSC, LUSC, and PAAD samples to classify prognosis as well as chronic diseases.

In four female carcinoma samples (COAD, HNSC, LUSC, and PAAD), three genes (AFAP1, NINJ2, and HOOK2) were commonly identified as classifiers, and the overexpression of the three genes was related to oncogenesis. The AFAP1 gene was hypermethylated in poor clusters rather than in good clusters. In the antisense of the AFAP1 gene, the AFAP1-AS1 gene has been known as an oncogenic long non-coding RNA in human carcinoma. AFAP1-AS1 was upregulated in lung cancer, and the function was revealed to promote invasion and metastasis [26]. The high expression of lncRNA AFAP1-AS1 promotes the progression of colon cancer and predicts poor prognosis [27].

The NINJ2 gene codes Ninjurin 2 protein, and its overexpression promotes human colorectal cancer cell growth in vitro and in vivo [28]. In the NINJ2 gene, rs118050317 polymorphism was related to endometrial cancer risk [29]. NINJ2 overexpression promotes glioma cell growth [30].

The limitations of this study are as follows. First, TCGA and KoGES samples were analyzed separately according to sex, but they were not separated according to age. Because carcinoma genesis is detected after the age of 50, the age ranges (40–69 years old) are similarly matched. Second, the bisulfite sequencing or array chip results were not confirmed by the experiment. Third, the TCGA and KoGES samples were derived from different sources except for TCGA-LAML. Fourth, TCGA has a cohort that mainly includes Westerners (it also includes Asians or other races), while the KoGES features a Korean cohort. There may be differences related to ethnicity, so there is a need to carry out similar methods in larger cohorts such as the UK Biobank. In the further studies, cross-validation experiments are needed in normal-cancer matched fresh blood samples in hospitals or community health centers.

Until now, research has been conducted to predict age based on DNA methylation patterns according to the “epigenetic clock” concept [15,16,31]. DNA methylation-based biomarkers have been developed to classify cancer and chronic diseases [9,10,17,25]. This study provides a new method of clustering by merging DNA methylation information from normal health check-up and TCGA samples. I presented a challenging method in this study and hope to discover more DNA methylation patterns that can distinguish disease samples from normal samples through similar research in the future. Since this study was conducted on Koreans, it needs to be merged with normal or diseased samples and experimentally verified in various countries in the future. Additionally, correlation studies between genotype data included in KoGES and TCGA and DNA methylation (DNA methylation quantitative trait locus; mQTL) are also needed. Recently, a study was published that predicted tumor response by integrating the three omics of TCGA [32], and another study investigated the relationship between GWAS result variants related to chronic diseases and somatic variants of TCGA [33]. In this way, disease-related patterns can be discovered through an appropriate combination of publicly available omics data.

In each country, the general public omics datasets will be accumulated and continue to be released, including the KoGES. By integrating omics datasets in an appropriate way, the value of omics datasets can be maximized to discover disease-related factors.

## 5. Conclusions

In this study, a method for the integrated analysis of public DNA methylation data was presented. Using the methodology of this study, CpG sites for cancer research can be extracted from TCGA, and chronic disease-related factors can be extracted from cohorts for normal subjects such as KoGES. The results of clustering in this study will be very helpful for predicting the patient’s prognosis and suggesting health management directions.

## Figures and Tables

**Table 1:** Commonly detected genes.

Names	No	Genes
COAD_F HNSC_F LUSC_F PAAD_F	3	AFAP1, NINJ2, HOOK2
HNSC_F LUSC_F LUSC_M PAAD_F	2	cg12036633, cg16814680
COAD_F LUSC_F LUSC_M PAAD_F	1	cg02159489
COAD_F HNSC_F PAAD_F	2	cg01156747, cg05528899
HNSC_F LUSC_F PAAD_F	6	MGMT, MRI1, UBAP2L, MYH15, cg27586797, cg21139150
HNSC_F LUSC_F LUSC_M	1	GNAS
HNSC_F PAAD_F	2	FBXL16, JRK
PAAD_F	8	RNF39, CCS, cg19214707, cg08103988, HLA-DRB1, HCG9, cg08779649, S100A14
HNSC_F	3	cg00456343, GDAP2, PMS2L11
COAD_F	2	NTNG1, PODN
LUSC_M	4	BCAT1, SOX2OT, KRT7, cg24000814

## Data Availability

All data were collected from public data sources. All source codes will provide as Supplementary Materials.

## Supplementary Materials

The following supporting information can be downloaded at https://www.mdpi.com/article/10.3390/life14060768/s1. Table S1: Full names of TCGA tumor types.

## References

[1] Liu J., Lichtenberg T., Hoadley K.A., Poisson L.M., Lazar A.J., Cherniack A.D., Kovatich A.J., Benz C.C., Levine D.A., Lee A.V. (2018). An integrated TCGA pan-cancer clinical data resource to drive high-quality survival outcome analytics. Cell.

[2] Tomczak K., Czerwińska P., Wiznerowicz M. (2015). Review The Cancer Genome Atlas (TCGA): An immeasurable source of knowledge. Contemp. Oncol./Współczesna Onkol..

[3] Network C.G.A.R. (2014). Comprehensive molecular characterization of gastric adenocarcinoma. Nature.

[4] Levine D.A. (2013). Integrated genomic characterization of endometrial carcinoma. Nature.

[5] Kim Y., Han B.-G., Group K. (2017). Cohort profile: The Korean genome and epidemiology study (KoGES) consortium. Int. J. Epidemiol..

[6] Cho H.W., Jin H.S., Eom Y.B. (2021). The interaction between FTO rs9939609 and physical activity is associated with a 2-fold reduction in the risk of obesity in Korean population. Am. J. Hum. Biol..

[7] Kim O.Y., Kwak S.-Y., Lim H., Shin M.-J. (2018). Genotype effects of glucokinase regulator on lipid profiles and glycemic status are modified by circulating calcium levels: Results from the Korean Genome and Epidemiology Study. Nutr. Res..

[8] Lee K.S., Rim J.H., Lee Y.-H., Lee S.-G., Lim J.-B., Kim J.-H. (2021). Association of circulating metabolites with incident type 2 diabetes in an obese population from a national cohort. Diabetes Res. Clin. Pract..

[9] Jung M., Ahn Y.-S., Chang S.-J., Kim C.-B., Jeong K.S., Koh S.-B., Gim J.-A. (2022). Variation in genotype and DNA methylation patterns based on alcohol use and cvd in the Korean genome and epidemiology study (KoGES). Genes.

[10] Ko Y.K., Kim H., Lee Y., Lee Y.-S., Gim J.-A. (2022). DNA Methylation Patterns According to Fatty Liver Index and Longitudinal Changes from the Korean Genome and Epidemiology Study (KoGES). Curr. Issues Mol. Biol..

[11] Moore L.D., Le T., Fan G. (2013). DNA methylation and its basic function. Neuropsychopharmacology.

[12] Greenberg M.V., Bourc’his D. (2019). The diverse roles of DNA methylation in mammalian development and disease. Nat. Rev. Mol. Cell Biol..

[13] Schmitz R.J., Schultz M.D., Urich M.A., Nery J.R., Pelizzola M., Libiger O., Alix A., McCosh R.B., Chen H., Schork N.J. (2013). Patterns of population epigenomic diversity. Nature.

[14] Link A., Balaguer F., Shen Y., Lozano J.J., Leung H.-C.E., Boland C.R., Goel A. (2013). Curcumin modulates DNA methylation in colorectal cancer cells. PLoS ONE.

[15] Gim J.-A. (2022). Integrative approaches of DNA methylation patterns according to age, sex, and longitudinal changes. Curr. Genom..

[16] Horvath S., Raj K. (2018). DNA methylation-based biomarkers and the epigenetic clock theory of ageing. Nat. Rev. Genet..

[17] Quach A., Levine M.E., Tanaka T., Lu A.T., Chen B.H., Ferrucci L., Ritz B., Bandinelli S., Neuhouser M.L., Beasley J.M. (2017). Epigenetic clock analysis of diet, exercise, education, and lifestyle factors. Aging (Albany NY).

[18] Colaprico A., Silva T.C., Olsen C., Garofano L., Cava C., Garolini D., Sabedot T.S., Malta T.M., Pagnotta S.M., Castiglioni I. (2016). TCGAbiolinks: An R/Bioconductor package for integrative analysis of TCGA data. Nucleic Acids Res..

[19] Leek J.T., Johnson W.E., Parker H.S., Jaffe A.E., Storey J.D. (2012). The sva package for removing batch effects and other unwanted variation in high-throughput experiments. Bioinformatics.

[20] Gomes R., Paul N., He N., Huber A.F., Jansen R.J. (2022). Application of Feature Selection and Deep Learning for Cancer Prediction Using DNA Methylation Markers. Genes.

[21] Sherafatian M., Arjmand F. (2019). Decision tree-based classifiers for lung cancer diagnosis and subtyping using TCGA miRNA expression data. Oncol. Lett..

[22] Clayton E.A., Pujol T.A., McDonald J.F., Qiu P. (2020). Leveraging TCGA gene expression data to build predictive models for cancer drug response. BMC Bioinform..

[23] Onwuka J.U., Li D., Liu Y., Huang H., Xu J., Liu Y., Zhang Y., Zhao Y. (2020). A panel of DNA methylation signature from peripheral blood may predict colorectal cancer susceptibility. BMC Cancer.

[24] Feng L., Jin F. (2018). Screening of differentially methylated genes in breast cancer and risk model construction based on TCGA database. Oncol. Lett..

[25] Koch A., Joosten S.C., Feng Z., de Ruijter T.C., Draht M.X., Melotte V., Smits K.M., Veeck J., Herman J.G., Van Neste L. (2018). Analysis of DNA methylation in cancer: Location revisited. Nat. Rev. Clin. Oncol..

[26] Zeng Z., Bo H., Gong Z., Lian Y., Li X., Li X., Zhang W., Deng H., Zhou M., Peng S. (2016). AFAP1-AS1, a long noncoding RNA upregulated in lung cancer and promotes invasion and metastasis. Tumor Biol..

[27] Bo H., Fan L., Li J., Liu Z., Zhang S., Shi L., Guo C., Li X., Liao Q., Zhang W. (2018). High expression of lncRNA AFAP1-AS1 promotes the progression of colon cancer and predicts poor prognosis. J. Cancer.

[28] Li G., Zhou L.-N., Yang H., He X., Duan Y., Wu F. (2019). Ninjurin 2 overexpression promotes human colorectal cancer cell growth in vitro and in vivo. Aging (Albany NY).

[29] Cheng Y., Yang L., Shi G., Chen P., Li L., Fang H., Chen C. (2021). Ninjurin 2 rs118050317 gene polymorphism and endometrial cancer risk. Cancer Cell Int..

[30] Zhou L.-N., Li P., Cai S., Li G., Liu F. (2019). Ninjurin2 overexpression promotes glioma cell growth. Aging (Albany NY).

[31] Horvath S., Gurven M., Levine M.E., Trumble B.C., Kaplan H., Allayee H., Ritz B.R., Chen B., Lu A.T., Rickabaugh T.M. (2016). An epigenetic clock analysis of race/ethnicity, sex, and coronary heart disease. Genome Biol..

[32] Ahn H.-M., Park I., Kim C.G., Ko Y.K., Gim J.-A. (2024). Factors related to tumor response rate from TCGA three omics data—Variants, expression, methylation. J. Environ. Sci. Health Part C.

[33] Jeon S., Park C., Kim J., Lee J.H., Joe S.-Y., Ko Y.K., Gim J.-A. (2023). Comparing variants related to chronic diseases from genome-wide association study (GWAS) and the cancer genome atlas (TCGA). BMC Med. Genom..

