# Supplementary material for: Survival Rate and Chronic Diseases of TCGA Cancer and KoGES Normal Samples by Clustering for DNA Methylation
Source: Life (Basel). 2024 Jun 17;14(6):768. doi: 10.3390/life14060768 (PMC11204879; doi:10.3390/life14060768)
Supplement: Supplementary file 1 [file life-14-00768-s001.zip › life-2929471-supplementary.pdf]

Table S1. Full names of TCGA tumor types.

Database name	Full name and tumor types
BRCA	Breast invasive carcinoma
COAD	Colon adenocarcinoma
HNSC	Head and Neck squamous cell carcinoma
LAML	Acute Myeloid Leukemia
LIHC	Liver hepatocellular carcinoma
LUAD	Lung adenocarcinoma
LUSC	Lung squamous cell carcinoma
PAAD	Pancreatic adenocarcinoma
PRAD	Prostate adenocarcinoma
STAD	Stomach adenocarcinoma
UCEC	Uterine Corpus Endometrial Carcinoma
